# Cell Death in AMD: The Rationale for Targeting Fas

**DOI:** 10.3390/jcm11030592

**Published:** 2022-01-25

**Authors:** David N. Zacks, Andrew J. Kocab, Joanne J. Choi, Meredith S. Gregory-Ksander, Marisol Cano, James T. Handa

**Affiliations:** 1 Department of Ophthalmology and Visual Sciences, Kellogg Eye Center, University of Michigan, Ann Arbor, MI 48105, USA; jochoi@med.wayne.edu; 2 ONL Therapeutics, Inc., Ann Arbor, MI 48104, USA; akocab@onltherapeutics.com; 3 Department of Ophthalmology, Schepens Eye Research Institute, Massachusetts Eye and Ear, Harvard Medical School, Boston, MA 02114, USA; meredith_gregory@meei.harvard.edu; 4 Wilmer Eye Institute, Johns Hopkins University, Baltimore, MD 21287, USA; mcano1@jhmi.edu (M.C.); jthanda@jhmi.edu (J.T.H.)

**Keywords:** macular degeneration, apoptosis, necroptosis, Fas

## Abstract

Age-related macular degeneration (AMD) is a leading cause of irreversible blindness in the developed world. While great advances have been made in the treatment of the neovascular (“wet”) form of the disease, there is still a significant need for therapies that prevent the vision loss associated with the advanced forms of dry, atrophic AMD. In this atrophic form, retinal pigment epithelial (RPE) and photoreceptor cell death is the ultimate cause of vision loss. In this review, we summarize the cell death pathways and their relation to RPE and retinal cell death in AMD. We review the data that support targeting programmed cell death through inhibition of the Fas receptor as a novel approach to preserve these structures and that this effect results from inhibiting both canonical death pathway activation and reducing the associated inflammatory response. These data lay the groundwork for current clinical strategies targeting the Fas pathway in this devastating disease.

## 1. Introduction

Age-related macular degeneration (AMD), a leading cause of irreversible blindness in the developed world, is a progressive disease affecting the central portion of the retina. The risk of developing AMD increases with age, and as the global population continues to grow older, the number of affected persons is predicted to steadily increase [1]. The exact pathogenesis of AMD is unclear, but a number of risk factors have been identified. As the name suggests, age is the most consistent risk factor for AMD, followed by environmental factors, such as cigarette smoking and high-fat diet. Genetic factors, such as gender, race, and polymorphisms in certain genetic loci, contribute to disease formation [2,3]. Under normal homeostatic physiology, the eye can tolerate these stressors; however, if sufficient stressors accumulate over time or with enough intensity, the compensatory protective pathways become overwhelmed, resulting in dysfunction of the retinal pigment epithelium (RPE) and buildup of cellular debris. Debris that accumulates under the RPE and retina is clinically apparent as drusen or reticular pseudodrusen, respectively. The size and density of these lesions are a hallmark of AMD progression [4]. In end-stage disease, RPE cells undergo programmed cell death, forming patches of RPE degeneration known as geographic atrophy (GA). RPE cell death subsequently leads to the loss of trophic support for the overlying photoreceptor cells that results in photoreceptor cell death. Loss of the RPE and photoreceptors ultimately leads to irreversible central vision loss. Thus, treatment that prevents GA formation or its progression is a clear, unmet need. Currently, many approaches are being tested, both pre-clinically and in patients, aimed at reducing GA lesion growth. Such approaches target various pathways implicated in AMD pathogenesis, including the complement system, mitochondrial stress, and the visual cycle, among others (reviewed in references [5,6,7]). In this work, we review cell death pathway activation during AMD and propose that targeting the Fas receptor is a novel approach to prevent RPE and photoreceptor cell death in atrophic AMD.

## 2. Overview of Programmed Cell Death

Historically, cell death has been classified into either apoptosis or necrosis. Apoptosis is a controlled form of cell death and “non-inflammatory”, as it does not activate a local inflammatory response characterized by macrophage infiltration [8,9,10]. In contrast, necrosis is typically thought to be an uncontrolled process that involves a large field of dying cells that induce a robust inflammatory response [9]. Although apoptosis and necroptosis result from activation of distinct molecular pathways, they share many morphologic characteristics, such as chromatin condensation, increased mitochondrial permeability, and DNA degradation [10]. No single test can unequivocally distinguish apoptosis from necrosis. Furthermore, aspects of necrosis that were thought to be passive and uncontrolled processes have since been associated with specific signaling pathways, indicating that under certain circumstances, necrosis may be more actively regulated than previously thought. With this updated insight, apoptosis and necrosis are now considered to exist as counterparts on the same continuum rather than being dichotomous opposites. To reflect these new nuances of cell death mechanisms, a new term was coined in 2005: necroptosis [11]. Like apoptosis, necroptosis is a form of regulated cell death but with key differences.

These two pathways have been extensively reviewed elsewhere [12]. Briefly, apoptosis is often divided into an intrinsic and extrinsic pathway. The intrinsic pathway is activated by insults, such as excessive reactive oxygen species (ROS), radiation, DNA damage, or oncogene activation. These cellular insults trigger cytochrome c release from the mitochondria, which activates a cascade of caspase cleavage and ultimately the degradation of cellular components. The extrinsic pathway is activated when ligands bind their respective transmembrane receptors in the tumor necrosis factor (TNF) superfamily, such as Fas, TNFR1, and TRAILR1. The receptors then oligomerize and recruit cytoplasmic factors that form the death-inducing signaling complex (DISC), which in turn activates additional downstream factors. The intrinsic and extrinsic pathways converge at caspase-3/6/7, which, along with other “executioner” caspases, lead to the degradation of intracellular substrates and ultimately cell death.

Members of the TNFR superfamily, such as Fas, which, upon trimerization, trigger DISC formation, can also activate the necroptotic pathway [13]. However, unlike apoptosis, necroptosis is independent of caspase activity and instead depends on receptor interacting protein kinases (RIPKs) to trigger a signaling cascade that activates membrane-damaging proteins and lytic cell death. It is the caspase-8 activation state that shifts the balance from apoptosis to necroptosis, as caspase-8 normally cleaves RIPK1 and RIPK3 and prevents its downstream effects. In contrast to apoptosis, necroptosis is inherently inflammatory due to its lytic nature. Despite the differences in the apoptotic and necroptotic molecular pathways, in practical application, no single test unequivocally distinguishes the two. Instead, both morphologic and biochemical data must be integrated to discriminate these two pathways [9,10,12].

While apoptosis and necroptosis are the most widely recognized pathways for regulated cell death, other cell death pathways exist [14]. In 2001, pyroptosis, a highly inflammatory form of regulated cell death, was described and initially observed in immune cells in response to intracellular pathogens [15]. Pyroptosis is initiated by the formation of an inflammasome, an intracellular multi-protein signaling complex that activates caspases and ultimately causes lytic cell death as a result of rapid plasma membrane pore formation [15]. The canonical pathway of pyroptosis relies on caspase-1 rather than apoptotic caspases-3/6/8. In the noncanonical pathway, caspases-4/5/11 are activated [15,16] Regardless of the pyroptotic pathway, the end result is the cleavage and activation of gasdermin D (GSDMD), particularly GSDMD-N, and the formation of pores in the plasma membrane that cause cell rupture. In 2012, ferroptosis was described [17]. Ferroptosis is a non-apoptotic pathway that relies on accumulation and overload of iron to generate ROS via the Fenton reaction [18]. Iron overload has been implicated in certain forms of retinal degeneration, including AMD, suggesting that ferroptosis may also play a role in this condition [19].

## 3. Programmed Cell Death in AMD

Apoptosis and necroptosis are historically thought to be the main mechanisms of cell death in AMD [20,21]. Among the vast body of AMD literature, most studies agree that RPE cells die by both necroptosis and apoptosis, whereas photoreceptors die primarily by apoptosis [22,23,24]. Early studies showed that photoreceptor and RPE cells in AMD eyes stained positive for DNA fragmentation (i.e., were positive for terminal deoxynucleotidyl transferase dUTP nick end labeling—TUNEL), suggesting activation of the apoptotic cell death pathway and also that the Fas/FasL pathway was the upstream regulator of apoptosis in AMD eyes [24].

More recently, evidence has emerged demonstrating that pyroptosis is also involved in RPE atrophy in AMD. Inflammasome activity and evidence of pyroptosis in AMD was first described in 2013 by Liu et al. and Tseng et al. [25,26]. In tissue sections of human eyes, histologic evidence of pyroptosis-like NLRP3 inflammasome activation was found in eyes of patients with GA or neovascular AMD [26]. RPE cells exposed to oxidative stress underwent cell death through the pyroptosis pathway if cells were grown in culture medium that primed inflammasome activation. Conversely, when caspase-1 activity was inhibited in these cells, death was decreased, suggesting that pyroptosis is a key pathway of RPE cell death in response to excessive oxidative stress [27]. An in-vivo study in rodents showed that a single intravitreal injection of amyloid beta (Aβ), a component of drusen, could induce a short-term inflammatory response in the RPE. Pyroptosis-specific markers, NLRP3 and caspase-1, were upregulated and peaked at four days post-injection, then dramatically subsided [25]. To build upon this work, subsequent studies attempted to mimic the chronic inflammation characteristic of drusen accumulation in aged human retinas. Aβ was injected over 14 days, which induced more robust NLRP3 inflammasome activity and elevated levels of cleaved caspase-1 [28]. Interestingly, evidence of both pyroptosis and apoptosis was seen in this study, which implicates that both pathways act in parallel during RPE cell death.

## 4. The Rationale for Targeting Fas

As mentioned above, therapy to prevent GA lesion formation and growth, which are the hallmarks of advanced dry AMD, is a large, unmet medical need. Currently, many approaches are being tested with the aim of reducing the growth of GA lesions. While some of these approaches have progressed to clinical testing, they have exhibited limitations, such as minimal reduction in disease progression and the need for frequent injections [29,30]. These factors suggest that alternative therapeutic targets are needed. Because the fundamental cause of vision loss in patients with dry AMD is the death of RPE and the overlying photoreceptors, it is logical to target the cell death machinery as a means to protect vision. Previously, several approaches have been taken (caspase inhibitors, etc.) with only limited success due to intracellular shunting of the death signal [5,31,32,33]. An alternative approach is to target the upstream points of the cell death signaling cascade activation. One potential target is the Fas receptor.

## 5. Fas Inhibition and Retinal Cell Protection

**Figure 1:**
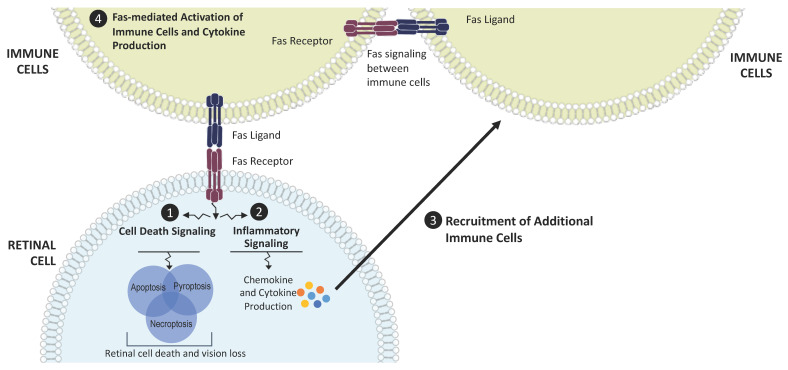
Fas as a regulator of cell death and inflammation in AMD. Disease stress leads to Fas activation in retinal cells and induces Fas signaling, which triggers (**1**) cell death signaling cascades and (**2**) production of cytokines and chemokines through the activation of inflammatory pathways. These molecules contribute to the inflammatory microenvironment and (**3**) recruit additional immune cells that bring more Fas ligand to the retina. These recruited immune cells also (**4**) activate nearby immune cells, leading to the production of additional inflammatory cytokines. This amplifies and propagates the inflammatory microenvironment and the cycle of retinal cell death and vision loss.

Fas is a transmembrane protein and a member of the tumor necrosis factor receptor gene superfamily [34]. Binding of the Fas ligand (FasL) to the Fas receptor activates the caspase cascade to induce cell death [35]. Activation of the Fas receptor also increases cytokine and chemokine production, leading to a pro-inflammatory state [36,37]. Fas receptor activation has been demonstrated in various models of retinal disease, such as retinal detachment, oxidative stress, and elevated intraocular pressure [38,39,40,41,42,43]. In these models, regardless of the stressor, the expression of Fas is increased; the caspase cascade is activated, typically demonstrated by the cleavage and activation of caspase 8; and cell death is increased. The cell death is accompanied by elevated cytokine and chemokine expression and increased retinal staining of Iba-1, a marker of microglia and macrophages. Thus, Fas activation represents a core, common pathophysiologic mechanism regulating retinal cell death across a variety of disease states (Figure 1).

In these various disease models, inhibition of Fas receptor activation reduces cell death and attenuates the inflammatory response [38,39,40,41,42,43]. Fas inhibition was accomplished both genetically and pharmacologically, the former through the use of the lpr mouse and the latter through the intravitreal administration of a variety of different agents. The lpr mouse strain contains a point mutation in the Fas gene, resulting in a non-functional protein that cannot activate the receptor’s downstream cascade upon binding with FasL. Pharmacologic inhibition in these models was achieved through intraocular injection of receptor antagonists, Fas neutralizing antibodies, inhibitory RNA that prevents Fas transcription, or an adeno-associated virus that results in the overexpression of an inhibitory, soluble form of the Fas ligand (heretofore called sFasL).

**Figure 2:**
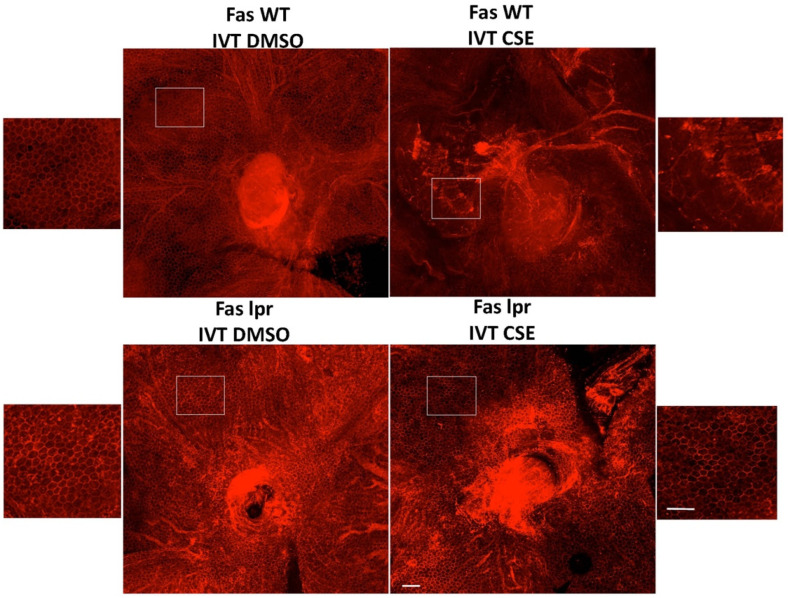
Fas deficiency protects the RPE from cigarette smoke extract-induced cell death. Wild-type control (Fas WT) or Fas-deficient lpr (Fas lpr) mice (*n* = 5 each) were given intravitreal CSE (250 µg/mL) in one eye or DMSO vehicle in the contralateral eye. After 10 days, RPE flatmounts were immunolabeled for ZO-1 to outline cell shape. The RPE of the wild-type mice given vehicle had preserved cobblestone morphology (**top left**), while the RPE of wild-type mice given CSE had disrupted ZO-1 labeling with ill-defined cell shape and enlarged, irregular contour (**top right**). In contrast, the RPE of lpr mice given either vehicle (**bottom left**) or CSE (**bottom right**) had regular cobblestone cell shape. Insets show magnified view of the RPE. Bar = 50 µm.

Work by Dunaief and colleagues demonstrated the marked upregulation of Fas in eyes from patients with macular degeneration [24], and Jiang and coworkers found an increase in soluble FasL in the serum of AMD patients [44]. To further assess the potential role of Fas inhibition in AMD, we used an acute cigarette smoke extract (CSE) model of RPE atrophy (Appendix A). Since cigarette smoking is a major risk factor for AMD development, this acute model mirrors a key aspect of the human condition [45,46]. CSE is a potent oxidative stressor and was injected into the vitreous cavity of the mouse eye to induce RPE cell death and atrophy. As can be seen in Figure 2, intravitreal injection of CSE in wild-type mice severely damages the normal RPE architecture as demonstrated by the massive disruption of the normal honeycomb pattern of the ZO-1 staining, a marker of RPE tight junctions.

To investigate the role of Fas in the RPE death in the CSE model, we first tested the model in the lpr mouse. The mouse, which, due to the point mutation in lpr, is a functional Fas receptor knockout, has structurally and functionally normal RPE and retina. We previously showed this strain was resistant to Fas-induced death of photoreceptors in experimental retinal detachment [39], RPE cell death in a model of oxidative stress [42], and retinal ganglion cell death in an experimental model of glaucoma [43]. In contrast to the effect of CSE in a normal mouse eye, intravitreal injection of CSE into the lpr mouse did not disrupt the RPE ZO-1 immunostaining pattern, consistent with the protection of the RPE from severe oxidative stress injury (Figure 2).

To therapeutically inhibit Fas, several modalities have been developed, including peptide inhibitors. In the sodium iodate model of AMD, RPE cell death is followed by subsequent death of the overlying photoreceptors, temporally similar to that observed in advanced atrophic AMD [42]. In that work, the investigators hypothesized that Fas receptor signaling contributes to RPE and photoreceptor death in the sodium iodate model of oxidative stress and blocked the action of the Fas receptor using a small peptide inhibitor called Met12, a 12-amino acid peptide derivative of the protein c-Met. In rats treated with Met12, the RPE and retinal structure were preserved after sodium iodate exposure compared to rats treated with an inactive scrambled peptide.

**Figure 3:**
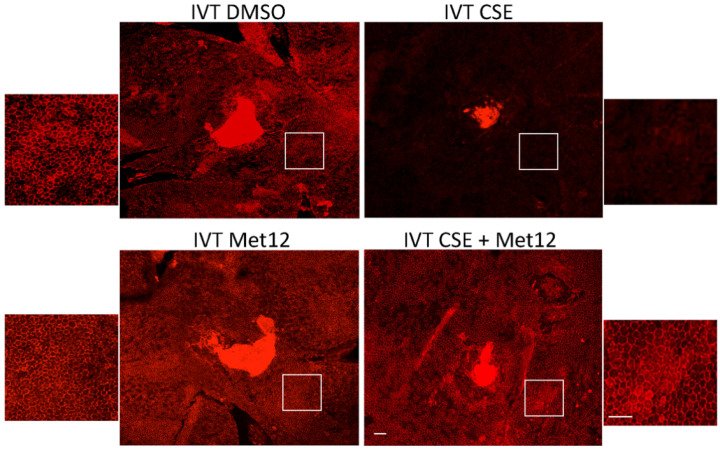
Peptide Fas inhibitor protects RPE from cigarette smoke extract-induced damage. Wild-type mice (*n* = 5 per group) were treated with the either DMSO (vehicle control) or the Fas inhibitor Met12 by intravitreal (IVT) injection and then IVT CSE 3 days later. After 10 days, RPE flatmounts were immunolabeled with anti-ZO-1. The RPE had a dramatic loss of hexagonal cell shape following IVT CSE (**upper right**). This was prevented with Met12 treatment (**bottom right**). White boxes highlight the location of the magnified images of the RPE. Bar = 50 µm.

**Figure 4:**
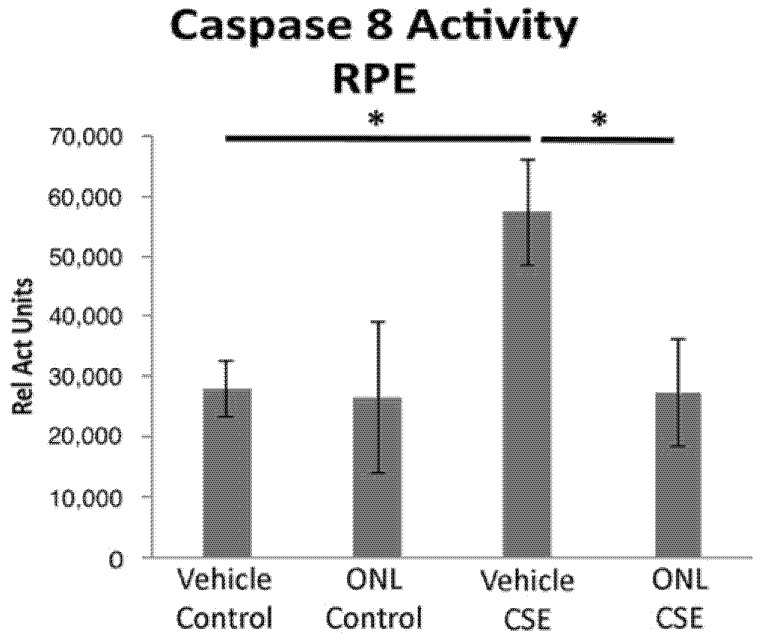
Peptide Fas inhibitor prevents caspase 8 activity in the RPE after cigarette smoke extract exposure. Wild-type mice (*n* = 5 per group) were treated with ONL1204 by intravitreal (IVT) injection in one eye and phosphate buffered saline (vehicle control) in the contralateral eye. Three days later, intravenous CSE 1000 μg/mL was given and then again 48 h later. After 3 days, the RPE was extracted to measure caspase 8 activity. The graph shows that caspase 8 activity is increased by CSE relative to control, and this is prevented by ONL1204; * *p* < 0.05.

When peptide Fas inhibitors were used in the acute CSE mouse model of dry AMD, similar protection was observed. In mice treated with a peptide Fas inhibitor, Met12, or its derivative ONL1204, RPE viability was preserved following challenge by CSE exposure (Figure 3). Furthermore, we measured the effect of the small peptide Fas inhibitor on the activation of caspase 8, the first downstream target of the activated Fas receptor. Reduction in caspase 8 activity levels is a direct measure of Fas inhibition. For this experiment, we used the derivative of the Met12 peptide, ONL1204. Due to its hydrophobic nature, Met12 can only be dissolved in DMSO, and this solvent interfered with the caspase 8 activity assay. ONL1204, on the other hand, is soluble in an aqueous buffer that did not interfere with the assay. As demonstrated in Figure 4, treatment with ONL1204 markedly reduced caspase 8 activation, reducing it to baseline levels. Collectively, these findings illustrate that inhibition of Fas activation by either genetic or pharmacologic methods results in marked RPE and neurosensory retinal protection in models of dry, atrophic AMD.

## 6. Fas Inhibition and Disease-Associated Inflammation

As with many ocular pathologies, inflammation has also been found to be involved in AMD pathogenesis [47]. Furthermore, significantly elevated concentrations of inflammatory cytokines have been detected in the serum and aqueous humor of patients with AMD [48,49], and higher levels of specific cytokines, such as IL-17, have been identified in the retina and choroid of donor eyes from patients with AMD [50]. Studies on the activation of the complement cascades in AMD have highlighted the role these pathways play in disease progression, and specific components of the complement pathways, including C3 and C5, have been identified in drusen from patients with AMD [51,52]. These findings underscore the contribution of inflammation in the progression of disease. Therefore, impacting the disease-related inflammatory microenvironment in parallel with protecting retinal cell viability may work synergistically to optimize treatment paradigms. Targeting Fas potentially represents an efficient means to accomplish this goal. Like its receptor relatives, Fas can activate inflammatory signaling and promote the inflammatory microenvironment through the production of inflammatory cytokines and chemokines, including IL-6, IL-8, MCP-1 (CCL2), CXCL-1, and sICAM-1 [53]. Mechanistically, Fas activation is thought to lead to the formation of a signaling complex in the cytoplasm comprised of FADD, caspase-8, RIP1, and cIAP1, which potentiates NF-κB-directed gene expression [37,53].

**Figure 5:**
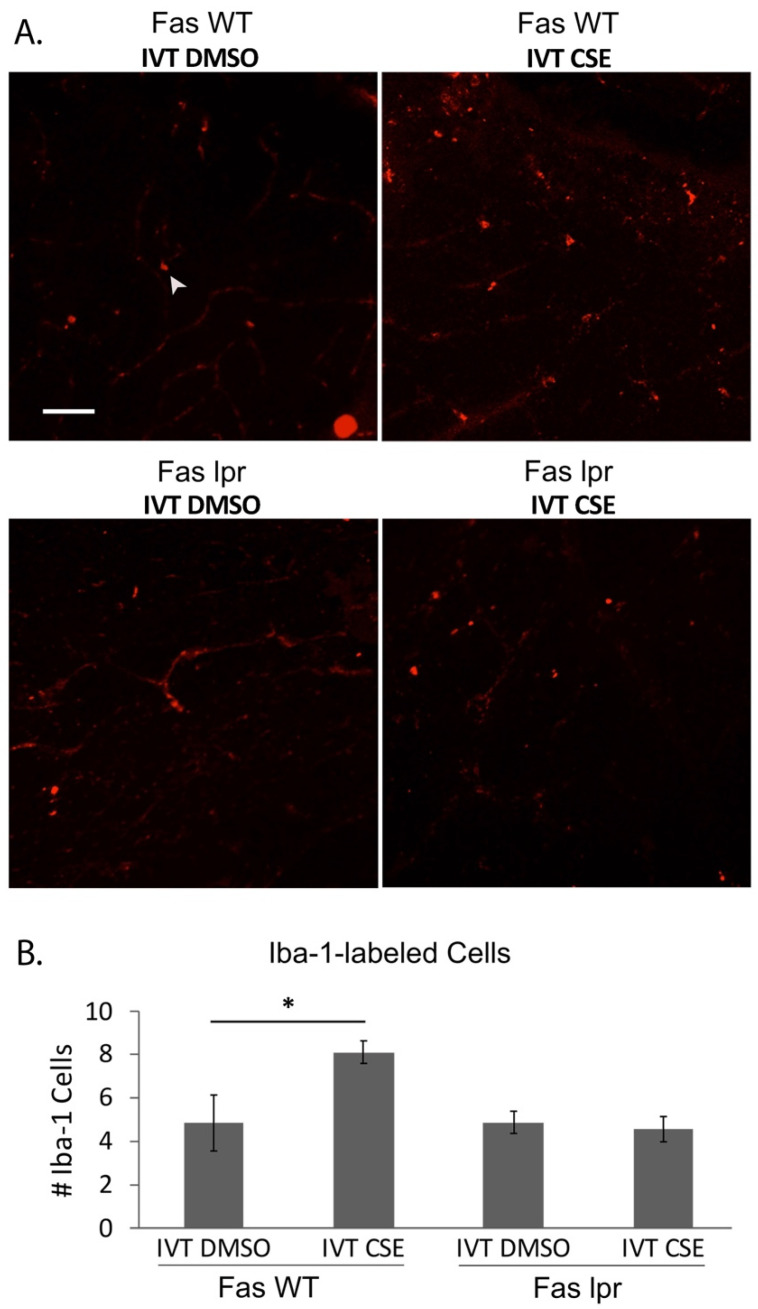
Fas deficiency reduces the inflammatory microenvironment following cigarette smoke extract. Wild-type control (Fas WT) or Fas-deficient lpr (Fas lpr) mice (*n* = 5 each) were given intravitreal CSE in one eye or vehicle in the contralateral eye. (**A**) After 10 days, retinal flatmounts were immunolabeled for Iba-1 to identify macrophages and microglia. Compared to control retina (**upper left panel**), the number of Iba-1-labeled cells (arrowhead) were increased after CSE stimulation (**upper right panel**), which was decreased in the retinas of lpr mice given either DMSO vehicle or CSE. Bar = 50 µm. (**B**) Graph quantifies the number of Iba-1-labeled cells in the retinas. * *p* < 0.05.

Correspondingly, Fas inhibition has been shown to prevent inflammation in the ocular microenvironment by us and others [42,43,54]. Furthermore, previous work from our group has shown that Fas inhibition can modulate the inflammatory microenvironment in animal models of AMD. In one study, the Met12 small molecule inhibitor of Fas reduced microglial activation in the sodium iodate model of RPE injury in the rat [42]. More recently, we expanded on this finding using the CSE model of RPE injury and atrophic AMD (Figure 5). In this model, Iba1-positive cells (microglia and macrophages) were significantly increased in the outer retina following intravitreal administration of CSE, consistent with the findings in the sodium iodate model and indicative of inflammation in the retina. Inhibiting Fas activation genetically with the lpr mouse or pharmacologically with Met12 resulted in significant reduction of the Iba1-positive staining, consistent with reduced activation of microglia and macrophages typically associated with this type of severe oxidative stress injury. Taken together, these data demonstrate that Fas inhibition, in addition to preserving cell viability, can reduce inflammation in animal models of RPE atrophy, highlighting the potential impact targeting Fas could have in a clinical setting.

## 7. Gene Therapy for Inhibiting Fas

While FasL binds Fas, the outcome of this interaction is dependent on the isoform of the ligand, whether membrane-bound or soluble. In 2000, Hohlbaum et al. suggested that the soluble form of FasL (sFasL) failed to activate inflammation characteristically observed in cells undergoing Fas-mediated apoptosis [55]. The difference between soluble and membrane-bound FasL was further demonstrated in glaucoma disease models. In mouse models of glaucoma, retinal ganglion cell (RGC) death and axon loss (a common endpoint for all forms of disease) was prevented in mice that received intravitreal injection of recombinant sFasL [56]. As opposed to the neuroprotective effects of sFasL, the effects of membrane-bound FasL (mFasL) were pro-inflammatory and pro-apoptotic. It was proposed that in the healthy eye, pro-inflammatory mFasL is normally cleaved to sFasL, the anti-inflammatory and neuroprotective form; however, in glaucoma, the balance of mFasL and sFasL shifts to favor the neurodestructive mFasL as the dominant form of FasL.

**Figure 6:**
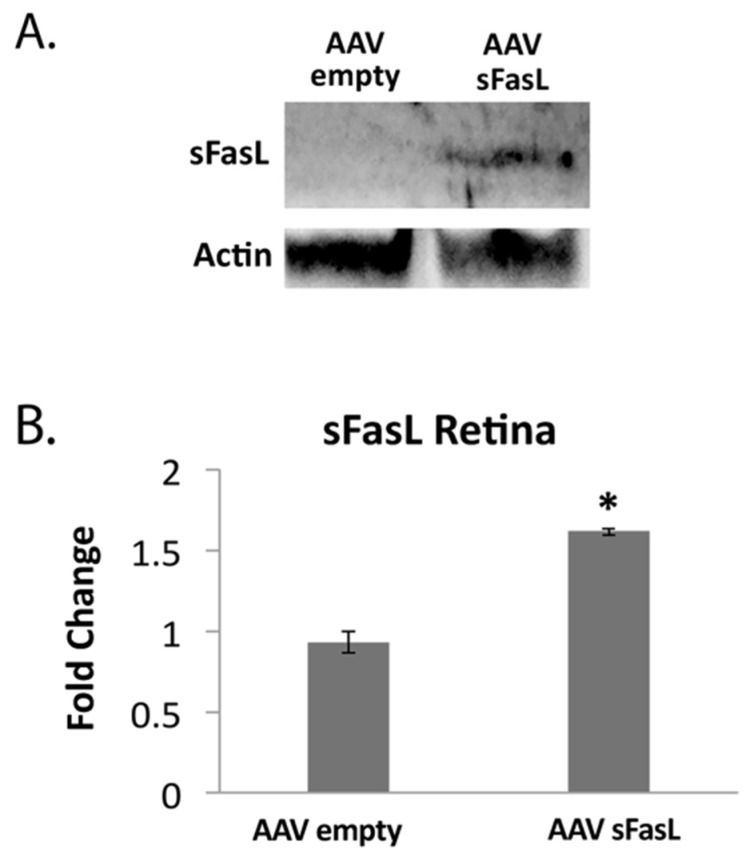
Measurable levels of sFasL are detected in the retina post IVT injection of AAV2-sFasL. Wild-type mice (*n* = 5 per group) were given IVT AAV2-sFasL or IVT AAV2-empty vector in each eye. (**A**) After 10 days, protein from neurosensory retinas was extracted for Western blot analysis using anti-sFasL. β-Actin was used for normalization. (**B**) Graph shows increased sFasL in retinas of mice that received AAV2-sFasL relative to mice receiving AAV2 empty vector. * *p* < 0.05.

**Figure 7:**
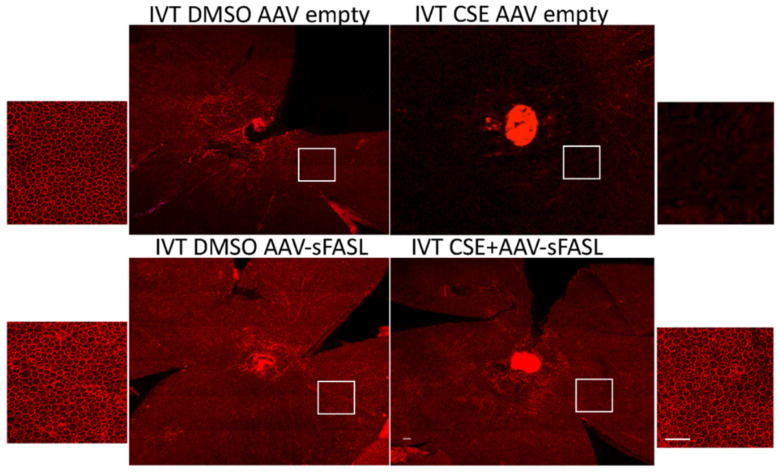
Fas inhibitor gene therapy AAV2-sFasL protects RPE from cigarette smoke extract-induced damage. Wild-type mice (*n* = 5 per group) were given IVT AAV2-sFasL or IVT AAV2-empty vector in each eye. Two weeks later, mice were given IVT CSE in one eye or DMSO vehicle in the contralateral eye. After 10 days, RPE flatmounts were immunolabeled with anti-ZO-1. The RPE lost its hexagonal shape after CSE injection. This damage was mitigated by AAV2-sFasL treatment. White boxes highlight the location of the magnified images of the RPE. Bar = 50 µm.

**Figure 8:**
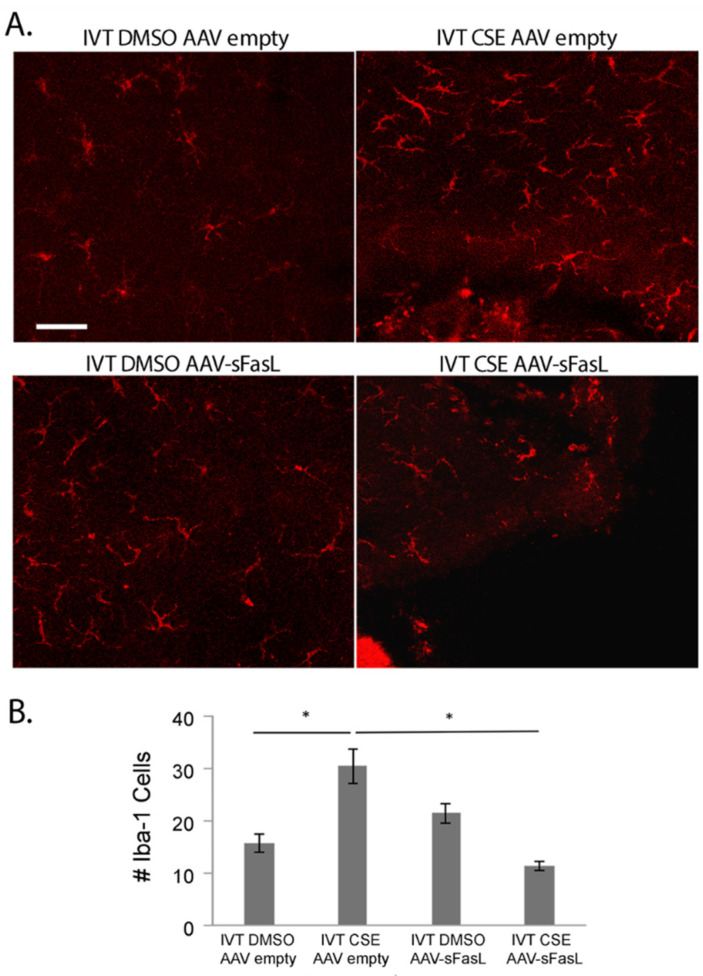
Fas inhibitor gene therapy AAV2-sFasL reduces the inflammatory microenvironment following cigarette smoke extract. Wild-type mice (*n* = 5 per group) were given IVT AAV2-sFasL or IVT AAV2-empty vector in each eye. Two weeks later, mice were given IVT CSE in one eye or DMSO vehicle in the contralateral eye. (**A**) After 10 days, retinal flatmounts were immunolabeled for Iba-1 to identify macrophages and microglia. Bar = 50 µm. (**B**) Graph showing that the number of Iba-1-labeled cells increases with CSE and that AAV2-sFasL decreases the number of Iba-1-labeled cells to control levels in CSE-treated mice. * *p* < 0.05.

To explore the long-term potential of inhibiting Fas activation as a therapeutic approach for treating dry AMD, an adeno-associated virus-mediated gene therapy approach was used to overexpress soluble Fas ligand (sFasL) in the acute CSE mouse model. Previous work demonstrated that a one-time intravitreal injection of AAV2-sFasL provided long-term expression of sFasL in the retina [57]. Moreover, this strategy of overexpressing sFasL in the retina has been shown to prevent Fas activation and subsequent loss of retinal ganglion cells in acute and chronic models of glaucoma [57]. As can be seen in Figure 6, the intravitreal injection of the AAV2-sFasL vector produced a measurable increase in sFasL protein detected in the retina. The true sFasL level, though, may actually be higher because, due to the secreted nature of the protein, we suspect that a significant percentage of the sFasL could not be captured by this assay. Figure 7 shows RPE flatmounts of control (empty vector) and AAV2-sFasL-treated eyes after intravitreal injection of CSE. As can be seen, the overexpression of sFasL preserved the normal RPE architecture despite CSE stress. This protection was also accompanied by a notable reduction in the CSE-induced increase in retinal Iba1-positive cells (Figure 8).

These data provide compelling evidence that blocking Fas receptor activation, genetically and pharmacologically, prevents RPE damage in the face of acute oxidative stress. Coupled with previously published reports, as described above, and the central role that Fas plays in the regulation of cell death, targeting Fas presents an attractive point of therapeutic intervention in atrophic AMD. Our findings suggest that Fas pathway inhibition should be explored as a novel method for treating GA.

## 8. Conclusions

AMD is a complex multifactorial disease for which there is currently no cure. While great advances have been made in the treatment of the neovascular (“wet”) form of the disease, there is still a significant need for therapies that prevent vision loss associated with the advanced forms of dry, atrophic AMD. Targeting programmed cell death through Fas receptor inhibition provides a novel approach of achieving this goal. Inhibiting this canonical death receptor provides a dual mechanism of action, inhibiting cell death directly in addition to reducing the inflammatory response associated with the disease process. Preclinical development of Fas inhibitors is currently underway with demonstration in chronic models of disease, and clinical trials will ultimately determine the utility of this approach.

## Data Availability

The data presented in this study are available on request from the corresponding author.

## Appendix A

### Materials and Methods

Mice: All experiments were conducted according to the ARVO Statement for the Use of Animals in Ophthalmic and Vision Research and were approved by the IRB at Johns Hopkins Medical School. Male and female wild-type (C57BL/6J; Jackson labs, Bar Harbor, ME, USA) MRL/MpJ-Fas^lpr^/J mice (lpr; Jackson labs), which are homozygous for the lymphoproliferation spontaneous mutation (fas^LPR^), and MRL/MpJ control mice (Jackson Labs) in a C57BL/6J background at 2 months old were used. Mice were given intravitreal injections (IVT) using a microinjection pump (Harvard Apparatus, Holliston, MA, USA).

Cigarette smoke extract and test articles: Cigarette smoke extract (CSE) challenge was performed as previously described [45,46]. For intravitreal (IVT) injection, the CSE was administered at a dose of 250 μL/mg in a volume of 1 μL of DMSO, and intravenous CSE was administered at a dose of 1000 μg/mL in a volume of 50 μL. Intravenous CSE administration was repeated 48 h later. Fas inhibitors were all administered via IVT injection. The following Fas inhibitors were used: ONL1204 (2 mg/mL in 1μL; ONL Therapeutics, Inc., Ann Arbor, MI), Met12 (10mg/mL in 1μL; ONL Therapeutics, Ann Arbor, MI), AAV2-sFASL (1 μL of 2 × 10^9^ gc/mL), or AAV2 empty vector (1 μL of 2 × 10^9^ gc/mL) (University of Massachusetts Chan Medical School Vector Core, Worcester, MA). At the appropriate time point, mice were euthanized, and eyes were enucleated. Tissue was processed for Western blot or flatmount immunofluorescence microscopy as previously described [45,46].

Flatmount immunofluorescence microscopy: Retina and RPE/choroid flatmounts were fixed in 2% paraformaldehyde overnight at 4 °C, rinsed with tris-buffered saline (TBS) that included 1% Triton × 100, and blocked with TBS/1% BSA overnight at 4 °C. RPE/choroid flatmounts were incubated with mouse anti-ZO1 monoclonal antibody (1:100; ZO1-1A12; Thermo Fisher Scientific, Waltham, MA, USA) and Alexa Fluor 594 (Thermo Fisher Scientific) overnight at 4 °C. Retina flatmounts were blocked with TBS/1% BSA and 5% goat serum overnight at 4 °C and incubated with goat polyclonal anti-AIF-1/IBA1 antibody (1:50; Novus biologicals, Centennial, CO, USA) for 48 h at 4 °C, washed, and incubated with donkey anti-goat Cy3 secondary antibody (Abcam, Cambridge, MA, USA) overnight at 4 °C. Flatmounts were examined with a confocal microscope (ZEN LSM 710, Carl Zeiss microscopy, Thornwood, NY, USA). Dendritic cells, defined by Iba-1-immunolabeled cell bodies with dendrites, were counted in five 20× power fields per flatmount.

Caspase 8 activity assay: Mouse retinas were harvested and protein was extracted as previously described [46]. The Luminescent Caspase-Glow 8 assay kit (Promega, Madison, WI, USA) was use according to manufacturer’s protocol.

Statistical significance, unless otherwise noted, was determined by one-way ANOVA with post-hoc Tukey–Kramer HSD testing.

Western blot analysis: Ten days after IVT injection of AAV2-sFasL, Western blot (WB) analysis was performed as described [46]. Membranes were incubated overnight with anti-FasL (1:10,000) at 4 °C and for 1 h with goat anti-rabbit HRP secondary antibody (1:1000; Abcam, Waltham, MA, USA). Immune complexes were visualized with SuperSignal West Pico chemiluminescent substrate (Pierce). Blots were imaged with an ImageQuant LAS4000 scanner (GE Healthcare, Inc., Piscataway, NJ, USA), and band intensity is reported as arbitrary densitometric units using ImageJ software. β-Actin (HRP conjugated, 643807, Biolegend, San Diego, CA, USA) was used for signal normalization. The statistical analysis was determined by Student’s unpaired *t*-test.
